# Homicidal Violence Against Women in Africa: Country-Level Trends From 1990 to 2021

**DOI:** 10.1177/08862605251353497

**Published:** 2025-07-15

**Authors:** Giloume Van Der Walt, Jason Bantjes, Ally Walker, Mohsen Naghavi, Dan J. Stein

**Affiliations:** 1 University of Cape Town, Cape Town, South Africa; 2 Institute for Health Metrics and Evaluation, Seattle, WA, USA

**Keywords:** gender-based violence, homicide, violence prevention, research methodology/measurement

## Abstract

Interpersonal violence against women (VAW), particularly femicide, remains a critical public health concern in Africa. Although age-standardized death rates have declined between 1990 and 2021, overall rates remain significantly higher than the global average. To better understand this issue, we conducted a descriptive analysis using modeled estimates from the Global Burden of Disease 2021 study to examine femicide trends across 54 African countries from 1990 to 2021. We assessed mortality using age-standardized mortality rates and morbidity using disability-adjusted life years. We found that mortality associated with physical violence involving sharp objects decreased over time, whereas no declines were observed in firearm-related or other forms of violence. Substantial variations emerged among countries, with some showing notable improvements and others experiencing worsening rates. Wide disparities in female-to-male homicide ratios underscored differing vulnerabilities across the continent. Moreover, the mean ages of female homicide victims varied considerably, pointing to possible age-specific risk factors. Despite some overall declines, femicide rates in Africa remain high, highlighting the need for context-specific strategies. Strengthening and localizing research efforts will help refine modeled estimates and enhance our understanding of femicide in Africa, thereby informing effective strategies to reduce VAW and improve their safety across the continent.

## Introduction

Interpersonal violence against women (VAW) remains a critical public health concern with profound social and developmental consequences. One of its most severe manifestations is femicide, broadly defined as the killing of females regardless of motive (Whittington et al., 2023). This inclusive definition encompasses all intentional killings of women and girls, providing a framework to analyze the prevalence and patterns of lethal violence across diverse contexts.

Globally, femicide persists at alarming levels. The United Nations Office on Drugs and Crime (UNODC, 2023) reported that approximately 88,900 females were killed worldwide in 2022. According to the Global Burden of Disease (GBD) study, which quantifies health loss by synthesizing multiple sources through statistical modeling, an estimated 70,290 women were killed globally in 2021 (IHME, 2024). In Africa, the number of female homicides increased from 12,570 in 1990 to 19,769 in 2021, highlighting a concerning upward trend. Although the age-standardized death rate for women declined—from 4.58 (95% CI [4.0, 5.2]) to 3.34 [2.8, 4.0] per 100,000—it remains nearly double the global average (1.76 per 100,000 in 2021).

VAW in Africa arises from complex sociohistorical and contemporary factors operating at multiple levels. The World Health Organization reports that 33% of women aged 15 to 49 in the African region have experienced intimate partner violence in their lifetime, with 20% in the past year, and 36% facing either partner or non-partner violence (WHO, 2021). Historically, Alesina et al. (2021) show that societies where women held active economic roles or where brideprice was customary exhibit lower male violence today. However, they also find that women’s economic independence can increase domestic violence risk, suggesting a backlash against shifting gender dynamics. Modern structural factors further exacerbate these patterns; Le and Nguyen (2022) document that a one standard deviation increase in nearby conflict raises intimate partner violence indices by 7% to 11%. At the institutional level, Okereke (2006) emphasizes how discriminatory laws, harmful traditions, and inadequate enforcement of gender-sensitive legislation perpetuate violence. These macro-level factors interact with local beliefs, as documented in Ghana, where research shows patriarchal notions that men “own” their female partners legitimize violence as a disciplinary mechanism (Sikweyiya et al., 2020).

While individual country studies reveal important determinants of violence, comparative epidemiological research documenting specific rates and patterns of femicide across Africa remains severely limited (Reis & Meyer, 2024). The UNODC (2023) notes that between 2010 and 2022, data on female homicides were available for at least 1 year in only 19 out of 58 African countries. A systematic review further underscores this issue, revealing that most femicide research within Africa is concentrated in South Africa (Variava & Dekel, 2024). This imbalance suggests that many African nations are underrepresented in the research, impeding a comprehensive understanding of regional variations, temporal trends, and specific types of gender-based lethal violence across the continent.

The GBD methodology attempts to address these limitations by prioritizing modeled estimates—with transparent uncertainty intervals—over the complete absence of data (Murray, 2022). This approach is crucial in countering the dangerous misconception that limited data equates to a minor problem, which frequently skews policy attention toward issues with stronger advocacy or data infrastructure. By integrating fragmented sources through advanced modeling techniques, GBD enables meaningful cross-country comparisons even in data-sparse settings, making it particularly valuable for analyzing estimates in contexts with limited primary data collection.

To begin addressing these gaps, our study analyses femicide trends and contextual factors across 54 African nations (1990–2021) using the 2021 GBD estimates. By examining death rates and disability-adjusted life years (DALYs) associated with VAW, the research seeks to provide nuanced insights into the temporal changes and geographical distribution of femicide in Africa. While acknowledging the limitations inherent in modeled data, the findings aim to contribute to the existing body of knowledge and support future research efforts toward enhancing women’s safety on the continent.

## Methods

This descriptive study analyzed modeled estimates from the GBD 2021 study to examine trends in femicide across 54 African countries from 1990 to 2021. Given the scarcity of primary data on femicide in many African countries, the GBD compiles data from various sources, including vital registration (VR) systems and verbal autopsy data. By employing advanced statistical modeling techniques, particularly the Cause-of-Death Ensemble Modelling framework, the GBD produces estimates that for a comprehensive assessment of the impact of interpersonal VAW across the continent.

Violence-related mortality estimates in Africa are challenged by the prevalence of garbage codes—nonspecific or implausible cause-of-death classifications that fail to accurately identify the underlying cause of death (Johnson et al., 2021). These codes emerge when deaths are attributed to intermediate causes, symptoms, or ill-defined conditions rather than specific underlying causes. While VR systems provide the highest quality cause-of-death data, these systems continue to be particularly insufficient in lower- and lower-middle-income countries where limited resources, insufficient physician training in proper certification practices, and weak health information infrastructure contribute to garbage code proliferation (Jha, 2014). The GBD addresses this challenge through empirical algorithms that redistribute garbage-coded deaths to plausible underlying causes based on statistical relationships between death patterns and relevant covariates. These algorithms employ multiple cause analysis, negative correlation approaches, and impairment-based redistribution techniques to reallocate misclassified deaths to more accurate causes, thereby providing more reliable estimates of violence-related mortality even in data-scarce environments (Johnson et al., 2021).

We focused on two primary outcome measures: the age-standardized mortality rate (ASMR) and DALYs attributable to interpersonal VAW. The ASMR represents the number of deaths due to interpersonal violence per 100,000 females, adjusted for age distribution. Age standardization is crucial for valid comparisons across countries with different demographic profiles, as it reduces variations due to differing age structures and minimizes potential confounding effects (Lopez et al., 2006). This adjustment provides a more accurate reflection of the mortality burden attributable to interpersonal violence among women in diverse populations.

DALYs quantify the total burden of disease by combining Years of Life Lost (YLLs) due to premature mortality and Years Lived with Disability (YLDs) from nonfatal health outcomes. These composite metric captures both fatal and nonfatal consequences, offering an integrated view of the impact of violence that extends beyond mortality alone. YLLs were calculated by multiplying the number of deaths at each age by the standard life expectancy for that age, adjusted for demographic variables such as age, sex, and location (GBD 2021 Diseases and Injuries Collaborators, 2024). YLDs were estimated by multiplying the prevalence of each nonfatal health outcome, or sequela, by its corresponding disability weight, accounting for age, sex, location, and year-specific prevalence (GBD 2021 Diseases and Injuries Collaborators, 2024). Together, YLLs and YLDs provide a holistic assessment of the health burden imposed by VAW.

Our analytical approach was designed to provide insights into the temporal changes and geographical distribution of femicide in Africa. We began by constructing a stacked bar chart illustrating annual ASMR from 1990 to 2021, segmented by types of interpersonal violence—specifically physical violence involving firearms, sharp objects, and other means. This visualization enabled us to assess temporal trends and shifts in the forms of violence contributing to female homicides over the 31-year period. To complement the visual data, we presented a corresponding table detailing the annual death rates and 95% uncertainty intervals for each type of violence.

To explore geographical variations, we developed four geospatial heat maps focusing on key metrics for 2021: the age-standardized death rates for females, the female-to-male death ratio, and the mean age of death by homicide for females. In addition, we mapped the variation in annual death rates from 1990 to 2021 for females across African countries, highlighting regions with significant changes over time. Furthermore, we compiled a data frame encompassing age-standardized death rates and DALY rates for females in 1990 and 2021, along with percentage variations over time. This data frame also included the variation in annual deaths, the mean age of homicide victims for both sexes in 2021, and the female-to-male death ratio for 2021. The female-to-male death ratio was calculated by dividing the total number of female deaths by the total number of male deaths for each country.

To calculate the mean age of homicide victims, we utilized the age categories provided by the GBD. The age groups used were <5 years, 5 to 9 years, 10 to 14 years, 15 to 19 years, 20 to 24 years, 25 to 29 years, 30 to 34 years, 35 to 39 years, 40 to 44 years, 45 to 49 years, 50 to 54 years, 55 to 59 years, 60 to 64 years, 65 to 69 years, and 70+ years. Each age category was assigned a midpoint value to approximate the average age within that range. For closed intervals (e.g., 5–9 years), the midpoint was calculated as the average of the lower and upper bounds. For the open-ended category “70+ years,” a midpoint of 75 years was assigned based on standard epidemiological practices. Using these midpoints, each age category was mapped to its corresponding numerical value. We then calculated the weighted deaths by multiplying the number of deaths in each age category by the respective midpoint age. This process was performed for each combination of location and sex. The data was subsequently grouped by location and sex to aggregate the total number of deaths and the total weighted deaths. The mean age of death was determined by dividing the sum of weighted deaths by the total number of deaths for each group. This method provided an estimated mean age of homicide victims, facilitating a comparative analysis across different countries and between genders.

All visualizations and analyses were conducted using Python (version 3.12.5) (Van Rossum & Drake, 2009) with essential libraries including pandas (McKinney, 2010) and numpy (Harris et al., 2020) for data manipulation and numerical operations, matplotlib.pyplot (Hunter, 2007) along with rcParams, TwoSlopeNorm, SymLogNorm, MaxNLocator, and FuncFormatter for creating and customizing visualizations, and GeoPandas for geospatial data handling and mapping. Shapefiles from the United States Department of State (United States Department of State, Office of the Geographer, 2013) were utilized for geospatial mapping.

## Results

**Figure 1.**
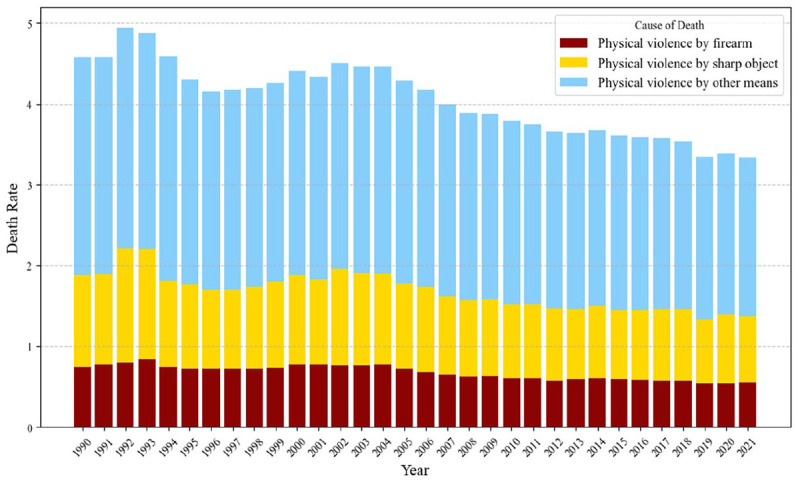
Stacked bar chart of age-standardized mortality rate (1990–2021) for homicide against females in Africa by violence. *Note.* This stacked bar chart represents the age-standardized mortality rate per 100,000 females in Africa from 1990 to 2021 by violence type.

Figure 1 presents a stacked bar chart illustrating the trends in ASMRs from different types of VAW in Africa between 1990 and 2021. Table 1 details these findings, showing a general decline in death rates across all homicide categories over the study period. Specifically, the ASMR for physical violence by firearm decreased from 0.74 (95% CI [0.60, 0.87]) per 100,000 population in 1990 to 0.55 [0.43, 0.73] in 2021; however, the overlapping confidence intervals suggest that this reduction may not be statistically significant. In contrast, deaths resulting from physical violence by sharp objects declined from 1.13 [0.99, 1.28] in 1990 to 0.82 [0.71, 0.95] in 2021, with non-overlapping confidence intervals indicating a statistically significant decrease. Similarly, physical violence by other means showed a reduction from 2.70 [2.31, 3.07] in 1990 to 1.97 [1.62, 2.42] in 2021, though the slightly overlapping confidence intervals also suggest that the decrease may not be statistically significant.

**Table 1.** Age-Standardized Death Rate Among Women in Africa by Homicide Type, 1990 to 2021.

Year	Physical violence by firearm	Physical violence by sharp object	Physical violence by other means
1990	0.74 (0.6, 0.87)	1.13 (0.99, 1.28)	2.7 (2.31, 3.07)
1991	0.78 (0.65, 0.88)	1.12 (0.99, 1.25)	2.69 (2.3, 3.04)
1992	0.8 (0.65, 0.92)	1.41 (1.27, 1.56)	2.74 (2.34, 3.1)
1993	0.85 (0.74, 0.96)	1.36 (1.15, 1.77)	2.68 (2.34, 3.0)
1994	0.74 (0.64, 0.85)	1.07 (0.96, 1.2)	2.77 (2.4, 3.21)
1995	0.72 (0.63, 0.82)	1.05 (0.95, 1.15)	2.54 (2.25, 2.82)
1996	0.73 (0.62, 0.84)	0.97 (0.86, 1.1)	2.46 (2.16, 2.77)
1997	0.73 (0.64, 0.83)	0.98 (0.87, 1.08)	2.48 (2.18, 2.76)
1998	0.72 (0.62, 0.82)	1.02 (0.91, 1.12)	2.46 (2.17, 2.73)
1999	0.73 (0.63, 0.84)	1.07 (0.96, 1.17)	2.46 (2.16, 2.75)
2000	0.77 (0.67, 0.86)	1.11 (1.0, 1.27)	2.52 (2.23, 2.81)
2001	0.77 (0.67, 0.88)	1.06 (0.95, 1.16)	2.51 (2.22, 2.82)
2002	0.76 (0.66, 0.86)	1.21 (1.1, 1.31)	2.54 (2.26, 2.81)
2003	0.76 (0.65, 0.86)	1.15 (1.05, 1.26)	2.56 (2.3, 2.82)
2004	0.77 (0.66, 0.87)	1.13 (1.02, 1.24)	2.56 (2.3, 2.82)
2005	0.72 (0.62, 0.82)	1.06 (0.96, 1.17)	2.5 (2.24, 2.79)
2006	0.69 (0.6, 0.77)	1.04 (0.95, 1.14)	2.45 (2.19, 2.73)
2007	0.66 (0.58, 0.74)	0.96 (0.87, 1.04)	2.39 (2.1, 2.66)
2008	0.62 (0.55, 0.71)	0.95 (0.86, 1.04)	2.32 (2.05, 2.61)
2009	0.63 (0.56, 0.72)	0.95 (0.85, 1.04)	2.3 (2.04, 2.62)
2010	0.61 (0.54, 0.7)	0.91 (0.82, 1.02)	2.26 (1.98, 2.6)
2011	0.6 (0.52, 0.71)	0.92 (0.82, 1.02)	2.22 (1.93, 2.59)
2012	0.58 (0.5, 0.69)	0.89 (0.8, 1.0)	2.19 (1.9, 2.56)
2013	0.59 (0.5, 0.71)	0.87 (0.77, 0.99)	2.18 (1.89, 2.58)
2014	0.6 (0.51, 0.73)	0.89 (0.8, 1.01)	2.18 (1.89, 2.58)
2015	0.59 (0.5, 0.73)	0.86 (0.76, 0.97)	2.16 (1.84, 2.57)
2016	0.58 (0.48, 0.72)	0.87 (0.76, 1.0)	2.14 (1.82, 2.54)
2017	0.57 (0.47, 0.74)	0.89 (0.77, 1.02)	2.12 (1.78, 2.56)
2018	0.57 (0.46, 0.73)	0.89 (0.78, 1.02)	2.08 (1.74, 2.52)
2019	0.54 (0.43, 0.71)	0.79 (0.68, 0.93)	2.02 (1.67, 2.47)
2020	0.55 (0.43, 0.72)	0.84 (0.73, 0.98)	1.99 (1.63, 2.44)
2021	0.55 (0.43, 0.73)	0.82 (0.71, 0.95)	1.97 (1.62, 2.42)

*Note.* Values represent the age-standardized homicide death rate per 100,000 women. Numbers in parentheses indicate the lower and upper bounds of the 95% confidence intervals (CIs).

**Figure 2.**
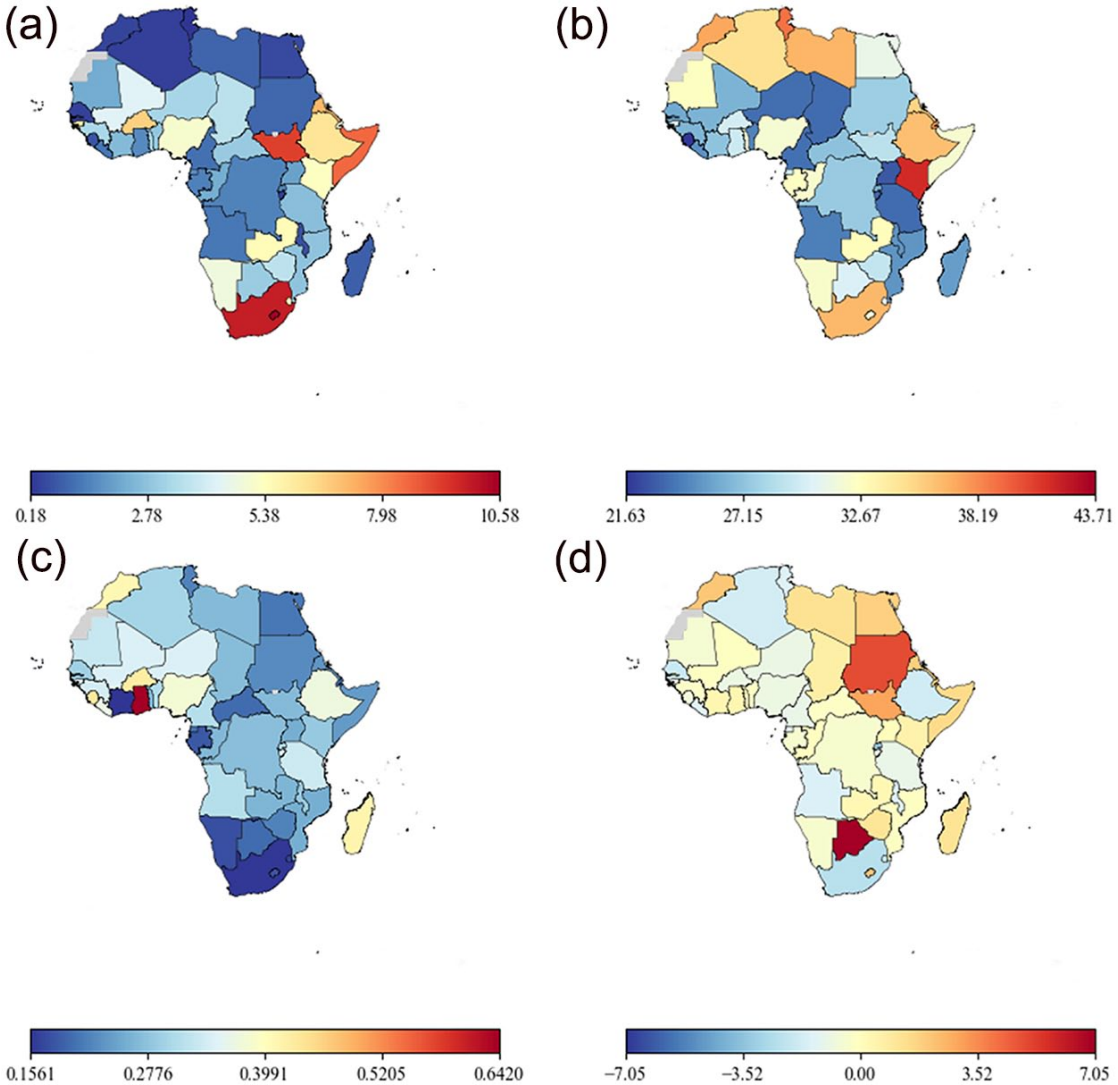
Geospatial heatmap of interpersonal violence indicators in Africa (2021) and trends from 1990 to 2021. *Note.* (a) Female age-standardized death rates in 2021; (b) Mean age of death for females in 2021; (c) Female/male death ratio in 2021; (d) Variation in annual deaths from 1990.

Figure 2 presents four geospatial heatmaps illustrating key indicators of homicide across Africa for the year 2021 and the trends from 1990 to 2021. Specifically, panel (A) displays the female age-standardized death rates, panel (B) shows the mean age of death for females, panel (C) illustrates the female-to-male death ratio, and panel (D) depicts the variation in annual death rates for females over the study period. Table 2 complements these visualizations by providing detailed country-level data corresponding to each of these indicators. These visualizations highlight regions with persistently high mortality rates and areas where significant changes have occurred, thereby aiding in the identification of high-risk areas. Significant disparities were observed at the country level.

**Table 2.** Country-Level Indicators of Interpersonal Violence in Africa (1990–2021).

Location	ASMR females 1990	ASMR females 2021	% Variation in ASMR from 1990 to 2021, females	% Variation in annual deaths from 1990 to 2021, females	Female/male death ratio 2021	2021 Male mean age of death	2021 Female mean age of death	Age-standardized DALY rate for females in 1990	Age-standardized DALY rate for females 2021	% Variation in DALY rate from 1990 to 2021, females
Algeria	0.66 (0.49, 0.86)	0.35 (0.26, 0.48)	−46.07 (−61.22, −20.88)	−1.71	0.30	32.88	34.76	92.96 (74.22, 113.03)	66.91 (53.72, 83.72)	−28.02 (−38.35, −17.03)
Angola	1.82 (1.35, 2.44)	1.30 (0.80, 2.06)	−28.66 (−58.99, 25.80)	−1.53	0.31	28.64	24.25	183.35 (145.77, 228.15)	139.54 (101.59, 199.36)	−23.89 (−44.94, 12.05)
Benin	2.67 (2.01, 3.34)	2.88 (1.91, 4.48)	8.09 (−30.67, 87.42)	−0.10	0.31	30.37	24.42	214.51 (170.29, 266.48)	213.71 (154.25, 305.49)	−0.37 (−29.42, 57.64)
Botswana	0.38 (0.26, 0.50)	3.01 (2.00, 4.28)	701.52 (382.88, 1170.80)	7.05	0.20	31.24	30.30	68.24 (54.81, 83.60)	231.00 (167.17, 304.96)	238.53 (146.58, 365.05)
Burkina Faso	8.55 (6.51, 11.16)	6.75 (4.48, 10.13)	−21.13 (−46.03, 17.18)	−0.88	0.43	36.02	29.91	459.11 (355.05, 587.62)	370.34 (255.41, 560.79)	−19.34 (−42.23, 20.11)
Burundi	0.36 (0.24, 0.50)	0.30 (0.16, 0.60)	−17.29 (−53.43, 59.29)	−0.85	0.34	28.38	23.43	85.88 (68.24, 110.37)	77.61 (58.04, 105.07)	−9.63 (−20.48, 9.76)
Cabo Verde	5.05 (3.69, 6.21)	3.87 (2.83, 5.04)	−23.35 (−47.87, 12.67)	−0.47	0.16	36.02	36.56	359.60 (273.00, 436.45)	263.09 (206.91, 330.80)	−26.84 (−46.17, 3.70)
Cameroon	1.56 (1.16, 2.21)	1.17 (0.67, 2.08)	−24.52 (−53.80, 28.45)	−1.04	0.31	28.04	23.74	154.99 (124.57, 198.43)	125.01 (89.81, 186.17)	−19.35 (−39.94, 15.69)
Central African Republic	2.93 (2.09, 3.82)	2.95 (2.06, 4.18)	0.84 (−32.51, 63.79)	−0.11	0.20	30.63	27.07	235.22 (181.73, 297.49)	236.42 (182.60, 314.44)	0.51 (−24.43, 46.93)
Chad	2.53 (1.88, 3.36)	3.63 (2.67, 4.90)	43.62 (−2.46, 107.11)	0.72	0.27	28.70	23.60	203.69 (160.63, 255.71)	255.23 (197.17, 328.55)	25.31 (−8.52, 71.16)
Comoros	3.78 (2.38, 5.19)	6.24 (4.71, 8.13)	65.16 (8.80, 185.39)	1.71	0.38	36.96	37.40	253.88 (164.61, 339.62)	351.35 (270.40, 456.34)	38.39 (−2.79, 125.86)
Congo	2.50 (1.27, 3.50)	2.32 (1.39, 3.39)	−7.18 (−39.49, 53.61)	0.00	0.27	32.96	32.12	208.59 (143.63, 272.30)	190.03 (140.72, 246.42)	−8.90 (−30.32, 26.24)
Côte d'Ivoire	2.13 (1.46, 3.10)	2.58 (1.60, 4.04)	20.86 (−20.66, 84.25)	0.43	0.16	36.60	27.18	173.34 (132.31, 231.73)	189.41 (131.45, 275.28)	9.27 (−19.30, 49.56)
Democratic Republic of the Congo	1.52 (1.01, 2.04)	1.52 (1.07, 2.15)	−0.11 (−34.98, 74.98)	−0.38	0.27	29.71	27.53	175.95 (137.73, 221.80)	164.36 (125.28, 215.49)	−6.58 (−26.95, 27.46)
Djibouti	2.91 (2.04, 4.47)	4.97 (3.25, 7.20)	71.00 (−0.75, 177.83)	1.59	0.20	36.59	37.88	229.94 (179.02, 318.12)	296.91 (215.96, 394.96)	29.13 (−11.18, 84.65)
Egypt	0.27 (0.24, 0.31)	0.51 (0.40, 0.63)	87.86 (48.05, 138.05)	1.84	0.21	31.69	31.05	61.52 (49.84, 74.45)	65.53 (53.40, 79.70)	6.51 (−3.61, 19.00)
Equatorial Guinea	2.33 (1.48, 3.22)	1.62 (0.72, 3.53)	−30.69 (−67.19, 64.86)	−1.25	0.20	29.07	29.31	207.04 (153.32, 269.50)	149.76 (96.25, 264.11)	−27.67 (−51.51, 32.40)
Eritrea	3.91 (2.84, 5.18)	7.28 (5.06, 9.88)	86.25 (24.15, 179.14)	1.89	0.23	33.21	35.42	277.43 (220.04, 353.45)	422.05 (308.92, 551.63)	52.13 (7.00, 119.97)
Eswatini	10.84 (8.78, 13.29)	5.50 (2.90, 9.28)	−49.25 (−72.21, −15.12)	−2.00	0.17	30.27	30.63	718.24 (600.85, 856.39)	362.90 (218.66, 573.23)	−49.47 (−68.98, −21.10)
Ethiopia	10.84 (6.74, 14.50)	6.24 (4.94, 7.99)	−42.47 (−61.64, 1.50)	−1.79	0.37	36.29	36.32	576.28 (379.72, 743.45)	329.48 (268.88, 402.15)	−42.83 (−60.36, −6.78)
Gabon	1.75 (0.85, 2.60)	1.47 (0.79, 2.79)	−15.88 (−53.29, 43.03)	−0.42	0.18	33.63	32.57	161.45 (109.88, 216.00)	136.46 (93.72, 218.01)	−15.48 (−39.55, 19.09)
Gambia	0.17 (0.12, 0.23)	0.18 (0.13, 0.29)	8.04 (−30.51, 83.73)	0.06	0.21	28.10	25.62	45.39 (36.90, 55.80)	38.13 (30.93, 47.10)	−16.01 (−25.86, −0.63)
Ghana	1.47 (1.11, 1.92)	1.63 (1.05, 2.41)	10.50 (−28.87, 74.78)	0.43	0.64	30.14	29.66	137.23 (113.58, 167.78)	139.95 (103.99, 190.63)	1.98 (−24.11, 42.17)
Guinea	2.93 (2.03, 3.98)	3.29 (2.25, 4.56)	12.10 (−30.22, 88.74)	−0.21	0.34	31.15	26.52	233.95 (171.73, 306.34)	233.77 (174.59, 308.76)	−0.08 (−31.97, 53.77)
Guinea−Bissau	7.58 (5.78, 9.87)	6.04 (4.09, 9.99)	−20.30 (−48.87, 30.48)	−0.93	0.34	31.59	30.32	498.48 (394.31, 633.85)	379.21 (274.04, 589.57)	−23.93 (−48.16, 22.37)
Kenya	4.87 (3.76, 6.87)	5.32 (4.07, 7.01)	9.21 (−21.78, 51.58)	0.57	0.28	36.81	41.72	294.56 (243.81, 366.82)	297.84 (241.90, 373.57)	1.11 (−19.05, 28.94)
Lesotho	5.58 (4.07, 7.21)	10.58 (6.72, 15.40)	89.51 (18.35, 193.60)	2.22	0.18	30.55	32.02	389.99 (301.78, 488.79)	664.68 (461.32, 904.79)	70.43 (16.02, 144.82)
Liberia	1.80 (1.40, 2.28)	1.27 (0.77, 1.98)	−29.39 (−56.53, 14.63)	−1.46	0.37	28.57	24.95	184.79 (153.86, 223.29)	129.20 (95.01, 178.44)	−30.08 (−47.98, −0.91)
Libya	0.84 (0.61, 1.15)	0.97 (0.59, 1.68)	14.88 (−31.19, 117.54)	1.30	0.27	33.57	36.73	98.52 (79.48, 122.44)	237.66 (190.33, 301.07)	141.23 (90.18, 214.53)
Madagascar	0.59 (0.45, 0.75)	0.90 (0.58, 1.49)	53.34 (0.83, 133.04)	1.25	0.42	27.69	25.46	101.95 (83.19, 124.18)	112.76 (85.82, 150.42)	10.60 (−8.15, 43.55)
Malawi	0.87 (0.62, 1.31)	0.64 (0.35, 1.30)	−26.38 (−56.06, 27.07)	−1.54	0.28	28.28	25.16	132.59 (103.06, 169.74)	117.63 (84.34, 167.11)	−11.29 (−25.42, 13.18)
Mali	3.94 (2.64, 5.21)	4.40 (3.28, 5.96)	11.66 (−23.93, 78.55)	−0.11	0.35	29.88	26.40	315.47 (227.72, 405.63)	309.88 (249.25, 392.77)	−1.77 (−27.52, 39.77)
Mauritania	2.42 (1.66, 3.18)	2.28 (1.65, 3.10)	−5.59 (−38.08, 62.92)	−0.45	0.33	33.82	32.51	180.95 (135.56, 222.35)	156.10 (120.91, 199.78)	−13.73 (−37.63, 30.79)
Mauritius	0.85 (0.77, 0.93)	0.52 (0.45, 0.59)	−38.49 (−47.51, −28.13)	−1.08	0.28	41.48	43.71	81.30 (71.27, 92.48)	60.92 (51.84, 71.38)	−25.07 (−31.31, −18.49)
Morocco	0.25 (0.19, 0.31)	0.46 (0.32, 0.66)	84.71 (27.80, 166.88)	2.11	0.41	35.17	37.28	67.87 (53.34, 83.45)	72.10 (56.71, 91.28)	6.24 (−5.41, 25.33)
Mozambique	2.39 (1.79, 3.11)	2.79 (1.70, 4.32)	16.72 (−27.14, 97.88)	−0.11	0.26	29.99	25.15	218.70 (174.44, 273.45)	219.88 (150.60, 318.89)	0.54 (−30.29, 57.27)
Namibia	5.85 (4.54, 7.53)	4.84 (3.02, 7.50)	−17.19 (−50.67, 35.37)	−0.35	0.18	31.46	32.22	385.62 (307.49, 474.64)	315.29 (216.08, 465.95)	−18.24 (−46.19, 28.39)
Niger	3.35 (2.26, 4.61)	3.24 (2.18, 5.21)	−3.13 (−41.38, 74.56)	−0.90	0.35	29.03	23.67	284.12 (205.09, 374.95)	238.91 (174.04, 342.98)	−15.91 (−44.41, 37.63)
Nigeria	6.03 (4.46, 7.84)	5.10 (3.30, 7.80)	−15.44 (−45.66, 40.68)	−0.85	0.38	32.26	31.93	349.25 (264.68, 439.29)	283.41 (196.01, 410.45)	−18.85 (−44.65, 29.39)
Rwanda	4.37 (3.59, 5.40)	1.50 (0.97, 2.35)	−65.66 (−77.75, −47.85)	−3.47	0.31	29.02	26.28	360.98 (303.48, 432.33)	159.65 (117.04, 218.47)	−55.77 (−67.26, −42.17)
São Tomé and Princípe	2.76 (1.84, 3.78)	2.07 (1.27, 3.58)	−24.77 (−53.28, 26.53)	−1.23	0.33	30.17	30.69	234.66 (175.21, 307.33)	169.81 (117.68, 267.08)	−27.64 (−47.81, 5.20)
Senegal	0.66 (0.49, 0.88)	0.41 (0.27, 0.64)	−37.99 (−58.70, −8.77)	−1.93	0.29	28.44	26.03	82.45 (67.61, 101.93)	59.91 (46.50, 78.08)	−27.34 (−39.78, −10.39)
Seychelles	2.31 (1.94, 2.75)	1.38 (1.09, 1.74)	−40.21 (−55.93, −19.30)	−1.31	0.31	41.91	42.23	170.88 (146.21, 197.52)	105.66 (86.85, 126.41)	−38.17 (−50.46, −22.92)
Sierra Leone	0.71 (0.47, 0.99)	0.68 (0.38, 1.25)	−3.34 (−42.74, 82.90)	−0.63	0.43	26.38	21.63	99.15 (79.55, 126.42)	82.90 (58.86, 120.72)	−16.39 (−37.50, 24.23)
Somalia	4.35 (2.82, 6.24)	8.56 (5.43, 14.87)	96.48 (24.56, 210.71)	1.53	0.24	31.48	31.99	315.50 (226.25, 425.95)	478.90 (329.14, 772.38)	51.79 (6.30, 124.07)
South Africa	22.43 (19.25, 25.10)	9.91 (8.54, 11.32)	−55.80 (−62.79, −48.40)	−2.47	0.16	33.55	36.60	1415.82 (1204.91, 1580.46)	597.51 (516.30, 683.22)	−57.80 (−63.47, −51.06)
South Sudan	3.24 (2.11, 4.88)	9.24 (6.80, 12.11)	184.89 (96.15, 332.40)	3.00	0.27	31.99	28.96	253.88 (181.95, 354.99)	550.27 (426.82, 698.81)	116.75 (61.58, 195.62)
Sudan	0.21 (0.17, 0.27)	1.02 (0.79, 1.40)	386.92 (272.46, 564.45)	4.98	0.23	28.64	27.71	67.60 (53.57, 84.29)	108.29 (87.84, 136.22)	60.19 (38.62, 98.68)
Togo	2.83 (2.20, 3.62)	3.07 (2.03, 4.60)	8.64 (−29.94, 64.20)	0.14	0.26	33.90	31.72	212.00 (171.18, 263.27)	208.39 (149.61, 302.73)	−1.70 (−30.00, 43.03)
Tunisia	0.38 (0.30, 0.48)	0.22 (0.14, 0.30)	−42.46 (−64.06, −11.06)	−1.47	0.22	35.79	39.09	72.22 (58.53, 88.38)	58.07 (45.07, 74.15)	−19.59 (−28.19, −10.99)
Uganda	1.83 (1.21, 2.71)	2.06 (1.20, 3.60)	12.43 (−33.70, 88.89)	0.08	0.26	27.39	22.89	210.66 (160.24, 276.66)	220.90 (159.94, 323.03)	4.86 (−21.03, 48.17)
United Republic of Tanzania	3.45 (2.74, 4.31)	2.66 (1.78, 3.93)	−22.81 (−49.03, 21.95)	−1.01	0.33	28.15	23.58	293.80 (239.03, 353.96)	235.92 (175.73, 318.38)	−19.70 (−40.20, 18.08)
Zambia	3.44 (2.74, 4.49)	5.53 (3.66, 7.87)	60.85 (6.46, 137.57)	0.30	0.26	33.75	32.84	303.59 (252.10, 370.58)	337.52 (242.16, 455.67)	11.17 (−17.54, 48.89)
Zimbabwe	2.85 (2.27, 3.64)	3.67 (2.48, 5.25)	28.64 (−14.76, 94.56)	1.15	0.22	29.71	29.25	225.76 (187.18, 272.77)	273.90 (206.73, 368.45)	21.32 (−9.80, 62.68)

*Note.* Values in parentheses indicate the lower and upper bounds of the 95% confidence intervals. Percentage variations represent relative changes from 1990 to 2021.

ASMR = age-standardized mortality rate per 100,000 population; DALY = disability-adjusted life years.

Several countries demonstrated substantial and statistically significant decreases in ASMRs and DALY rates, indicating progress in reducing female homicides. For instance, South Africa experienced a remarkable decrease in ASMR, declining by 55.8% (95% CI [−62.8, −48.4]), from 22.4 deaths per 100,000 [19.2, 25.1] in 1990 to 9.9 [8.5, 11.3] per 100,000 in 2021. Correspondingly, its DALY rate decreased by 57.8% [−63.5, −51.5], from 1,415.8 [1,204.9, 1,580.5] per 100,000 to 597.5 [516.3, 683.2] per 100,000 in the same period. Rwanda also showed significant improvements, with its death rate declining by 65.7% [−77.7, −47.8], from 4.4 [3.6, 5.4] per 100,000 in 1990 to 1.5 [1.0, 2.4] per 100,000 in 2021, and its DALY rate decreasing by 55.8% [−67.3, −42.2], from 361.0 [303.5, 432.3] per 100,000 to 159.7 [117.0, 218.5] per 100,000. Other countries with notable reductions include Algeria (ASMR reduction of 46.1%, [−61.2, −20.9]), Eswatini (49.3% reduction, [−72.2, −15.1]), and Tunisia (42.5% reduction, [−64.1, −11.1]).

Conversely, some countries exhibited dramatic increases in mortality and DALY rates, highlighting worsening conditions. Botswana experienced the most significant surge, with its ASMR increasing by 701.5% [382.9, 1,170.8], from 0.4 [0.3, 0.5] per 100,000 in 1990 to 3.0 [2.0, 4.3] per 100,000 in 2021, and its DALY rate increasing by 238.5% [146.6, 365.0], from 68.2 [54.8, 83.6] per 100,000 to 231.0 [167.2, 305.0] per 100,000. South Sudan’s death rate rose by 184.9% [96.2, 332.4], from 3.2 [2.1, 4.9] per 100,000 to 9.2 [6.8, 12.1] per 100,000, and its DALY rate increased by 116.7% [61.6, 195.6], from 253.9 [182.0, 355.0] per 100,000 to 550.3 [426.8, 698.8] per 100,000. Other countries with significant increases include Sudan (ASMR increase of 386.9%, [272.5, 564.4]), Lesotho (89.5% increase, [18.4, 193.6]), Eritrea (86.3% increase, [24.2, 179.1]), and Egypt (87.9% increase, [48.0, 138.1]).

Countries with the highest female-to-male homicide death ratios include Ghana (0.64), Sierra Leone (0.43), and Burkina Faso (0.43). Conversely, countries with the lowest ratios are Côte d’Ivoire (0.16), South Africa (0.16), and Cabo Verde (0.16). These ratios indicate that in Ghana, Sierra Leone, and Burkina Faso, the proportion of female homicide victims relative to males is higher compared to countries such as Côte d’Ivoire, South Africa, and Cabo Verde, where female homicides are significantly less prevalent relative to male homicides.

The mean age of homicide victims varies between genders and across countries. For males, the highest mean ages were observed in Seychelles (41.91 years), Mauritius (41.48 years), and Comoros (36.96 years). For females, the highest mean ages were found in Mauritius (43.71 years), Seychelles (42.23 years), and Kenya (41.72 years). In contrast, the lowest mean ages for male homicide victims were in Sierra Leone (26.38 years), Uganda (27.69 years), and Madagascar (27.69 years), while for females, the youngest mean ages were recorded in Sierra Leone (21.63 years), Uganda (22.89 years), and Burundi (23.43 years).

Significant discrepancies exist between the mean ages of male and female homicide victims in several African countries. Côte d’Ivoire exhibits the largest disparity, with male victims having a mean age of 36.6 years compared to 27.18 years for female victims, resulting in a 9.43-year difference. Burkina Faso also shows a notable gap, where the mean age for male victims is 36.02 years, while female victims average 29.91 years, creating a discrepancy of 6.11 years. Benin presents a similar pattern, with male victims having a mean age of 30.37 years compared to 24.42 years for females, leading to a 5.95-year difference. These pronounced age disparities suggest that younger women may be more vulnerable to homicide compared to their male counterparts in these countries.

## Discussion

This study provides a comprehensive analysis of homicide trends among women in Africa from 1990 to 2021, revealing both encouraging progress and persistent challenges in addressing violence across the continent. The ASMR for female homicides declined overall, with deaths from physical violence by sharp objects showing a statistically significant reduction from 1.13 (95% CI [0.99, 1.28]) to 0.82 (0.71, 0.95] per 100,000. Declines in firearm-related homicides (0.55 per 100,000 in 2021) and other means were not statistically significant, as indicated by overlapping confidence intervals. These findings rely on GBD’s modeled estimates, which, while robust, may introduce uncertainty in data-sparse regions, necessitating cautious interpretation pending further primary data collection.

When compared to Brazil, where Pinto et al. (2022) examined VAW aged 15 to 49 years from 1990 to 2019, Africa demonstrates relatively lower rates of certain types of homicide, although methodological differences, such as age standardization, should be considered. Specifically, firearm-related homicides against women in Africa were 0.55 (95% CI [0.43, 0.73]) per 100,000 in 2021, substantially lower than Brazil’s 4.0 (3.8, 4.3] per 100,000 in 2019. Similarly, deaths due to physical violence by sharp objects were lower in Africa (0.82 per 100,000 in 2021) compared to Brazil (1.8 per 100,000 in 2019). In the United States, the overall female ASMR was 2.42 [2.33, 2.52] per 100,000 according to GBD estimates (IHME, 2024), but with a significantly higher firearm-related homicide rate of 1.45 [1.39, 1.51] per 100,000. Other sources report even higher rates; for instance, a study using data from the Centers for Disease Control and Prevention found 2.0 fatalities per 100,000 women due to firearm-related homicides in the United States in 2021 (Rees et al., 2022). It is important to note that these figures were not age-standardized, which may account for some differences in rates. This stark contrast highlights the differing nature of lethal VAW in different regions and suggests that firearm-related interventions may be more critical in other settings than in Africa.

Significant heterogeneity exists across African nations. South Africa achieved a 55.8% reduction in ASMR (95% CI [−62.8, −48.4]), from 22.4 [19.2, 25.1] to 9.9 [8.5, 11.3] per 100,000, yet its 2021 rate remains high compared to other African countries. Rwanda also showed progress, with a 65.7% decline [−77.7, −47.8], from 4.4 [3.6, 5.4] to 1.5 [1.0, 2.4] per 100,000, potentially linked to post-conflict recovery and gender equality policies, though direct causation cannot be confirmed without further research. Conversely, Botswana experienced a 701.5% increase [382.9, 1,170.8], from 0.4 [0.3, 0.5] to 3.0 [2.0, 4.3] per 100,000. Despite this relative surge, Botswana’s absolute rate remains lower than South Africa’s, underscoring the need to balance relative and absolute metrics when prioritizing interventions. Algeria, Eswatini, and Tunisia also reported reductions (46.1%, 49.3%, and 42.5%, respectively), possibly reflecting legislative advancements, such as Tunisia’s 2017 Law 58 on gender-based violence (UNDP, 2018a) and Algeria’s 2015 domestic violence law (UNDP, 2018b). However, contradictions, such as Algeria’s Penal Code Article 279 allowing leniency for spousal homicide in adultery cases, highlight persistent legal barriers.

The female-to-male homicide death ratios varied across countries, with higher ratios observed in Ghana (0.64), Sierra Leone (0.43), and Burkina Faso (0.43), and lower ratios in Côte d'Ivoire (0.16), South Africa (0.16), and Cabo Verde (0.16). While male homicide rates are higher than female rates in all countries, the relatively higher female-to-male ratios in some nations suggest that women are at a comparatively higher risk relative to men in those settings compared to others. This may reflect societal norms, gender inequalities, cultural practices that increase women’s vulnerability, or differences in reporting and data collection. For instance, higher ratios may indicate prevalent intimate partner violence or harmful traditional practices that disproportionately affect women. Further research is needed to understand the factors contributing to these variations.

The analysis of the mean age of homicide victims revealed notable differences between genders and across countries. In some countries, female victims were significantly younger than male victims. For example, in Côte d'Ivoire, the mean age of male victims was 36.6 years compared to 27.18 years for female victims, a difference of 9.43 years. Similar patterns were observed in Burkina Faso and Benin, with differences of 6.11 and 5.95 years, respectively. While mean age data should be interpreted with caution—since outliers and a high number of deaths in very young or very old age groups can affect the results—the consistent age differences suggest that younger women may be particularly vulnerable to homicide in these countries.

Although identifying precise causal factors remains challenging given the continent’s diverse socioeconomic and political contexts, there are indications that a confluence of developmental and preventive strategies may be contributing to these observed overall declines in VAW. According to the UNODC (2019), socioeconomic development and targeted violence-prevention programs are often associated with reductions in violent crime. In addition, the WHO (2014) points to enhanced policing strategies, strengthened legislation, and community-based interventions as potentially influential in curbing homicide rates.

Potential explanations for high mortality rates among younger women may include intimate partner violence and gender-based violence targeting younger age groups. Yet, such risks can emerge even in infancy. Research conducted in South Africa indicates that infant abandonment is significantly higher in girls than in boys (Mathews et al., 2013). In that study, 35.5% of the recorded child deaths were perpetrated by an acquaintance, and 43% were cared for by a single mother. Notably, 45.4% of the murdered girls from their sample were killed by their mother. While these findings are specific to South Africa and may not be generalizable to all African countries, they highlight critical risk factors associated with child homicides. Conversely, older women face distinct risks, potentially tied to societal neglect, accusations of witchcraft, or cultural practices that increase vulnerability (de Jong & Pokwana ka Menziwa, 2022; Machangu, 2015; Owusu, 2023). These age-specific vulnerabilities highlight the need for interventions addressing VAW across the lifespan, from infancy to old age, while acknowledging the context-specific nature of these risks.

Understanding the multifaceted risk factors driving VAW is critical for effective interventions in Africa, yet their interrelated and context-specific nature complicates isolating single causes. Socioeconomic status plays a complex role; while lower household wealth is often linked to higher VAW (Ahmadabadi et al., 2020; Jewkes, 2013; Mojahed et al., 2022), research across sub-Saharan Africa shows violence persists across all wealth levels (Bamiwuye & Odimegwu, 2014). Early marriage heightens vulnerability to IPV and domestic violence (Gubi et al., 2020; Nyato et al., 2019), and relationship dynamics, such as male infidelity, can act as a catalyst (Pichon et al., 2020). Education is generally protective, with higher levels associated with lower violence perpetration and victimization (Amegbor & Pascoe, 2021; Conroy, 2014). However, this relationship is not universal; some studies suggest educated women may still face violence due to entrenched societal norms (Peterman et al., 2011; Weitzman, 2018). Botswana illustrates this paradox: despite significant educational gains for both genders (World Bank, 2023), its female homicide rates surged, indicating that education alone cannot mitigate VAW without addressing structural and cultural factors, such as those rooted in historical gender norms (Alesina et al., 2021).

Societal acceptance of violence remains a fundamental barrier to progress against gender-based violence in Africa (Alio et al., 2011; Boyle et al., 2009; Uthman et al., 2009), exacerbating the structural challenges noted in the introduction. This creates a complex environment where interventions must simultaneously target individual risk factors and deeply embedded cultural norms. Our epidemiological analysis reveals intricate VAW patterns, but the intervention landscape remains significantly limited, particularly in Africa, where evidence on prevention strategies lags behind high-income regions (Ellsberg et al., 2015). Most programs focus on response rather than prevention, with limited evaluation of their effectiveness or sustainability.

These findings underscore an urgent need to transform how VAW is addressed in Africa. The dramatic variations in mortality rates demand context-specific primary research to identify vulnerable populations and predominant causes in each setting. VAW manifests differently across the continent, affecting diverse age groups, involving varied forms of violence, and occurring at disparate rates. Interventions effective in one country—such as Tunisia’s legislative reforms (UNDP, 2018a)—may not succeed in another with different structural challenges, such as police corruption or weak enforcement. Economic interventions like microfinance and property rights reforms may be critical where poverty increases vulnerability, whereas community-based programs challenging harmful gender norms might better address cultural acceptance of violence. Effective policies must be grounded in rigorous epidemiological data and theory-informed models that address violence across the social ecology (Michau et al., 2015). Sustainable solutions require locally developed, cost-efficient strategies designed for systemic transformation, rather than imported frameworks. Coordinated action that translates epidemiological insights into tailored prevention strategies—respecting local contexts while learning from regional successes—is essential to protect women’s fundamental right to safety across Africa.

## Limitations

The reliance on modeled data from the GBD study introduces inherent uncertainties, particularly in regions such as Africa, where empirical data are often limited or unavailable. Many African countries lack comprehensive VR systems and robust data collection infrastructure, leading to potential underreporting or misclassification of femicide and interpersonal violence. Cultural norms, stigma, fear of retaliation, and mistrust in legal systems can discourage victims and communities from reporting incidents of VAW, contributing to the underestimation of true homicide rates.

While age-standardized data facilitate comparisons across the continent, they may not capture the nuanced distribution of mortality rates across different age groups and countries. This could obscure critical insights necessary for formulating precise policy measures and interventions. In addition, the study did not account for intranational regional variations. Significant disparities may exist between urban and rural settings, regions with varying levels of economic development, or areas experiencing conflict versus those that are stable. Generalizing findings to entire national populations without considering these regional differences may result in misleading conclusions and less effective policy recommendations.

In addition, while we present comprehensive descriptive trends, our analysis does not incorporate significant country-specific socioeconomic, political, and cultural variables that might explain the observed patterns. This primarily descriptive approach provides valuable documentation of trends but offers limited insight into the specific mechanisms driving these patterns. Compounding these methodological constraints is a substantial literature gap across much of Africa, with existing research disproportionately concentrated on South Africa. We acknowledge these limitations while emphasizing that establishing reliable baseline measurements across the continent represents not only a necessary first step toward more explanatory research but also an initial contribution toward addressing the critical knowledge deficit in this field.

## Conclusion

This study provides valuable insights into the trends and patterns of homicide against women in Africa from 1990 to 2021. While there has been an overall decline in age-standardized death rates, significant disparities persist across countries and age groups. Some nations have made notable progress in reducing female homicide rates, whereas others have experienced increases, highlighting the complex and heterogeneous nature of VAW on the continent. The variations in female-to-male homicide ratios and the mean ages of victims suggest that cultural, social, and legal factors play critical roles in shaping these trends. Our findings underscore the need for targeted, context-specific interventions to reduce homicide rates among women and enhance their safety. However, because these patterns rely in part on modeled estimates, further research—including localized data collection and methodological refinements—will be essential to more accurately inform and tailor violence-prevention strategies across Africa.
